# Outcomes from Indo–United States–Egypt tri-national psychiatric research training programmes

**DOI:** 10.1186/s12961-020-00595-9

**Published:** 2020-07-17

**Authors:** Tulsi A. Malavia, Vishwajit Nimgaonkar, Triptish Bhatia, Ibtihal M. A. Ibrahim, Hader Mansour, Maribeth Wesesky, Joel Wood, Smita N. Deshpande, Mary Hawk

**Affiliations:** 1 University of Pittsburgh School of Medicine, 3550 Terrace Street, Pittsburgh, PA 15213 USA; 2 Psychiatry and Human Genetics, Program for Genetics and Psychosis, School of Medicine and Graduate School of Public Health, University of Pittsburgh, Pittsburgh, PA USA; 3 Indo-US Projects, Department of Psychiatry, Centre of Excellence in Mental Health, ABVIMS - Dr. Ram Manohar Lohia Hospital, Bangabandhu Sheikh Mujib Road, New Delhi, 110001 India; 4 Mansoura Faculty of Medicine, Mansoura University, Mansoura City, Egypt; 5 Addiction Medicine Services Inpatient Unit, Western Psychiatric Institute and Clinic, University of Pittsburgh School of Medicine, Pittsburgh, PA USA; 6 Department of Psychiatry, University of Pittsburgh, Pittsburgh, PA 15213 USA; 7 Department of Psychiatry, Centre of Excellence in Mental Health, ABVIMS - Dr. Ram Manohar Lohia Hospital, Bangabandhu Sheikh Mujib Road, New Delhi, 110001 India; 8 University of Pittsburgh Graduate School of Public Health, 6120 Public Health, 130 DeSoto Street, Pittsburgh, PA 15260 USA

**Keywords:** Psychiatric research, programme evaluation, LMICs

## Abstract

**Background:**

The prevalence of mental health disorders is increasing globally. Countries in South Asia, Southeast Asia and the Middle East regions carry high burdens of mental health need; however, there are relatively few mental health research publications from this region, suggesting inadequate research funds and a paucity of qualified research personnel. To increase and strengthen the pool of mental health researchers in India and Egypt, we conducted three psychiatric research programmes in these countries: the Training Program for Psychiatric Genetics in India (2002–2011), the Tri-National Training Program for Psychiatric Genetics (2009–2014) and the Cross-Fertilized Research Training for New Investigators in Egypt and India (2014–2019). A total of 66 trainees, including psychiatrists, psychiatric social workers, clinical psychologists and research psychologists, were supported in research development, which included didactic training, proposal development, hands-on research and manuscript preparation.

**Methods:**

The aim of this study is to evaluate these three training programmes using the four-level Kirkpatrick Model of Training Evaluation that assesses reaction, learning, behaviour and outcomes. A descriptive analysis was used to explore the data collected throughout the duration of the three training programmes. Online surveys were crafted and sent to the mentors and trainees of the three programmes to supplement objective training data.

**Results:**

In addition to positive changes in the areas of reaction, learning and behaviour, significant outcomes were demonstrated. As of the writing of this manuscript, the trainees published a total of 130 papers, 59 as first author. In addition, 26 trainees have co-authored papers with one or more trainees or mentors, which demonstrates successful research networking and collaboration.

**Conclusion:**

Our findings suggest that our training approach is a successful model for building independent mental health researchers. This is a critical step in the development of effective mental health interventions in low- and middle-income countries.

## Background

The prevalence of mental disorders has increased in low- and middle-income countries (LMICs) partly due to their rapidly growing populations and increases in life expectancy. However, the prevalence of mental disorders is increasing globally as well [1, 2]. In 2014, 322 million people were estimated to have depression worldwide, equating to roughly 4.4% of the global population – this reflects an 18.4% increase since 2005. In 2015, 264 million people worldwide were estimated to have some form of anxiety disorder, comprising approximately 3.6% of the world population, reflecting a 14.9% increase since 2005 [3]. The global prevalence of schizophrenia and bipolar disorder is over 23 million and 60 million, respectively [4]. The Southeast Asia and Western Pacific regions bear high burdens of mental health issues, partly due to their large populations [5]. Approximately 50% of people with depression worldwide live in these regions. Additionally, Southeast Asia is the region with the highest prevalence of anxiety disorders [3]. As the prevalence of mental illnesses continues to rise, so does the global burden of disease [6–9]. This is especially problematic given that mental disorders such as schizophrenia lack effective treatment and treatment reach, further increasing the global burden [10, 11]. With increasing prevalence and a high burden of psychiatric disorders on the global population, psychiatric research is essential.

Although psychiatric disorders are prevalent worldwide, with the highest rates in Eastern countries like India and China, these countries lack representation in psychiatric research publications. Six leading psychiatric journals were surveyed to determine the country of origin of their published research manuscripts from all issues in the years 1996–1998. Of these journals, 94% of the published papers originated in Western Europe, North America, Australia or New Zealand. Thus, only 6% of research papers originated from the rest of the world, even though it accounted for 90% of the global population [12]. These findings were replicated in a second study by the same author examining manuscripts published from 2002 to 2004 [13]. During 1998 and 2008, 90% of peer-reviewed research publications in the top 10 psychiatric journals originated from countries in the North American, Northern European, Oceanic and Western European regions even though these countries account for only 10% of the world population [14]. This discrepancy could be due to a relative disparity in research funds or to unavailability of qualified research personnel in the rest of the world.

The global burden of psychiatric disorders as well as the lack of psychiatric literature being produced from Eastern countries underscores the need for training local researchers in developing countries. Psychiatric research training programmes that educate new investigators to perform high-quality psychiatric research in LMICs are needed [15]. In order to improve the capacity of psychiatric researchers in LMICs and ultimately reduce the burden of disease, we conducted and reviewed three psychiatric research training programmes – the Training Program for Psychiatric Genetics (TPPG) in India (2002–2011), the Tri-national Training Program for Psychiatric Genetics (TNTPPG) (2009–2014) and the Cross-Fertilized Research Training (CFRT) for New Investigators in Egypt and India (2014–2019). Additionally, while the CFRT was ongoing and directly because of need highlighted during this work, two workshops on grant writing were conducted. The first workshop was a collaborative effort with the Indian Council of Medical Research (ICMR), held in November 2016 [16] and the second workshop was conducted in conjunction with the Indian Association of Private Psychiatry (IAPP) and the United States National Institute of Health (NIH) in February 2018. The evaluation of the three training programmes and two workshops were conducted by an external evaluation team consisting of a medical student and a faculty mentor from the University of Pittsburgh Graduate School of Public Health.

### The TPPG in India

The TPPG was a non-degree programme based in Delhi, India, and Pittsburgh, PA, United States, with five trainees who were motivated candidates from psychiatry and allied specialties. Two post-doctoral trainees were long-term, whose training spanned 5 years. Three post-graduate trainees were short-term, who participated in didactic training activities for 3 months. All trainees were assigned a mentor in the United States and in India.

The first 1-year phase of the long-term, post-doctoral training comprised of didactic learning. During this year, both long-term trainees stayed in Pittsburgh for mentorship in practical research skills and education on psychiatric genetics, genetic epidemiology and ethics in research with human subjects. Didactic training included training modules and classes at the University of Pittsburgh. Trainees completed an R21-style research proposal, an NIH developmental research grant award for the early stages of project development and received funding from the training programme to complete a research project. Research projects were focused on schizophrenia, bipolar disorder and major depression. The second phase was field training and research, which lasted 2.5 years. During this time, the trainees returned to New Delhi, India, whereupon they initiated their research projects. Throughout this period, the trainees continued to receive distance education and training from their mentors in the United States as well as from their co-mentors in India. The third phase of long-term training lasted 6 months, when the trainees returned to the United States to analyse the data from their research and began writing their manuscripts as well as subsequent grant applications. The fourth and final phase of the project lasted 1 year and incorporated re-integration into local research in Delhi. During this year, trainees were supported in accessing independent research funding and commenced collaborative research with their mentors.

The TPPG also supported three short-term trainees. These candidates were PhD students in India who visited mentors in the United States for 1–3 months of didactic training. They learned research skills, psychiatric genetics, ethics in research with human subjects and developed sample R21 research protocols. These protocols were subsequently funded by the programme for 2-years of research. All three short-term trainees completed their graduate research and were awarded PhDs.

### The TNTPPG

The TNTPPG, conducted nationally with trainees from India and Egypt, was a degree-awarding programme with four long-term trainees – two from India and two from Egypt. All four trainees completed their PhDs from universities in their home countries. Trainees were matched with a local mentor and an international mentor. The duration of the programme was of 3 years. The first year was didactic training, during which the trainees came to the United States for 3–6 months to learn practical research skills and obtain education on research ethics and psychiatric genetics. The research projects from this programme focused on schizophrenia or autism. At the end of this phase, trainees wrote and submitted their PhD theses based on their research training to their universities.

### CFRT for new investigators in Egypt and India

The third training programme was also implemented nationally in Egypt and India and incorporated medium-term and long-term trainees. The medium-term trainees completed a 6-month training programme during which they were paired with a mentor, completed 4 months of didactic coursework and developed their own research protocols. The didactic coursework involved a series of 13 online lectures, which include an ethics module as well as pre- and post-tests. The long-term trainees were required to do the same didactic coursework as the medium-term trainees; however, instead of developing a research protocol, they developed and completed projects for their PhDs from universities in their home countries. These research protocols and projects focused on interventional treatments for schizophrenia. All long-term trainees were paired with international mentors.

### Collaboration with the ICMR

During the implementation of the CFRT in India, the ICMR requested a 1-week capacity-building workshop to provide grant writing support to psychiatric researchers in India. This workshop was conceived as a ‘Grantathon’, a sprint-style, week-long workshop in which trainees from diverse backgrounds receive mentorship from international researchers with the goal of developing fundable proposals addressing mental health needs by the end of the week. During this capacity-building workshop, participants were provided information relating to proposal development, research and evaluation design, and collaboration development. The Grantathon workshop was conducted in 2016. Mentors from India and Egypt advised mid-level faculty members from Indian psychiatric institutions (*n* = 24) to develop 12 single- or multi-site research grant applications [16]. These applications were subsequently funded by the ICMR.

### IAPP–NIH training grant workshop

During the first three training programmes, requests were received for research training support from members of non-governmental organisations. In response to these requests, a 1-day workshop was organised in February 2018 with the help of the IAPP. This was a smaller version of the earlier Grantathon [16] and targeted members of IAPP as well as private mental health professionals interested in research. The objective of the workshop was to provide hands-on training to members of the IAPP (which included mid-level faculty from the psychiatry departments or related departments from medical colleges and universities) to develop viable research proposals that could be submitted for funding. Participating psychiatrists presented their ideas and were guided to write the main aim of their studies focusing on psychiatric research.

We report on findings of the three training programmes and two workshops, evaluation results based on the Kirkpatrick Model of Training Evaluation [17, 18]. The retrospective evaluation included three parts – analysing data collected throughout the training programmes, supplementing data with additional surveys and determining the degree to which programme aims were met. The broad goals of the study were to assess opportunities for programme improvement and to determine the training impact on research capacity in the target regions.

## Methods

The evaluation framework was built on the Kirkpatrick Model of Training Evaluation [17, 18], which incorporates four levels of training assessment – reaction, learning, behaviour and results. Reaction (Level 1) assesses the overall opinions and subjective responses of the training programme from the participants’ perspectives. Learning (Level 2) refers to the attainment of specific knowledge, skills and attitudes gained by training participants. Level 3, behaviour, assesses the transfer of knowledge into practice, which is critical for independence in research. The fourth and final level of the Kirkpatrick Model of Evaluation is the results level, which evaluates the overall success of the training programmes.

### Level 1: Reaction

To assess trainees’ subjective experiences in the training programme we conducted surveys using Qualtrics Online Survey Software [19]. Descriptive analyses were used to analyse survey results. Research trainees were asked about how useful they found the training programme, how much contact they had with their mentors during their active training periods, how useful their mentors had been in their development as independent researchers, and the strengths and weaknesses of the programme. All responses were anonymous.

In addition, all trainees answered questions regarding their opinion on the ethics module and their mock R21 research protocol or PhD proposal. Trainees from the TPPG and TNTPPG were asked about their opinion of the classes they took at the University of Pittsburgh. Trainees in the CFRT Grant were asked about the lecture videos they watched for the didactic training. They indicated how many hours they spent watching the videos, which videos were most beneficial to them, and whether they thought the pre- and post-lecture quizzes accurately represented the content in the videos.

### Level 2: Learning

To assess trainees’ learning we organised and analysed the data collected as part of standard practices within the training programme. These included 66-item pre- and post-test scores from online didactic lectures that were accessed by trainees in the CFRT. We analysed changes in these scores using a paired samples *t*-test. Trainees in the other programmes were asked to assess their own changes in learning via the Qualtrics online survey that asked about the benefit of the courses taken at the University of Pittsburgh, the ethics module they completed and the R21 style grant they wrote.

### Level 3: Behaviour

All trainees were asked to identify how the programme benefitted their careers and how their current research corresponded to the research proposed or performed during their training [20]. All survey data were collected anonymously by an independent evaluator not funded through the training grant programmes.

### Level 4: Results

As the primary goal of the training programmes was to train independent researchers in mental health research, Level 4 was assessed by monitoring the number of publications secured by trainees. The total number of publications, citations and collaborations in co-authorship are reported below.

## Results

A total of 66 trainees were supported across the three training programmes and the IAPP–NIH workshop. Of these, 37 (56%) were women and 29 (44%) were men (Table 1).

**Table 1:** Participant demographics

	Male		Female		Total
The Training Program for Psychiatric Genetics in India	2	(40%)	3	(60%)	5
Tri-National Training Program in Psychiatric Genetics (Egypt and India)	2	(50%)	2	(50%)	4
Cross-Fertilized Research Training Grant (India and Egypt)					
Medium-term Class 1	5	(45%)	6	(55%)	11
Medium-term Class 2	6	(46%)	7	(54%)	13
Medium-term Class 3	7	(50%)	7	(50%)	14
Medium-term Class 4	4	(40%)	6	(60%)	10
Long term	1	(25%)	3	(75%)	4
Indian Association of Private Psychiatry^a^	2	(40%)	3	(60%)	5
	29	(44%)	37	(56%)	66

^a^Indian Council of Medical Research ‘Grantathon’ participants have been described in Hawk et al. [16]

### The TPPG in India

Five people were trained in the TPPG from 2002 to 2011. All the trainees were from India and completed the Qualtrics survey. Table 2 shows participants’ assessments of the training programme, stratified by training group and the Kirkpatrick Levels of Training Evaluation.

**Table 2:** Number of participants indicating ‘strongly agree’ or ‘agree’ in assessing value of training modules, by training group and Kirkpatrick levels of training evaluation

		TPPG ***n*** = 5	TNTPPG ***n*** = 4	CFRT (in progress) ***n*** = 30^**a**^
Level 1: Reaction	Value of training programme	5 (100%)	4 (100%)	28 (93%)
	Value of mentors	5 (100%)	4 (100%)	22 (73%)
Level 2: Learning	Value of proposal development	5 (100%)	4 (100%)	24 (80%)
	Value of ethics module	5 (100%)	4 (100%)	22 (73%)
	Value of didactic coursework	4 (80%)	3 (75%)	NA^b^
Level 3: Behaviour	New job or better research position	3 (60%)	2 (50%)	5 (17%)
	Collaboration with mentors	4 (80%)	2 (50%)	14 (47%)
	Collaboration with trainees	1 (20%)	2 (50%)	7 (23%)
	Training alignment with research	4 (80%)	4 (100%)	17 (57%)
	Deepened interest in psychiatric genetics	3 (60%)	2 (50%)	17 (57%)
Level 4: Outcomes	Publications	5 (100%)	4 (100%)	10 (33%)

*CFRT* Cross-Fertilized Research Training, *NA* not available, *TNTPPG* Tri-National Training Program in Psychiatric Genetics, *TPPG* The Training Program for Psychiatric Genetics

^a^Number of trainees completing the surveys

^b^Questions not asked of this training group

#### Level 1: Reaction

All five trainees found the programme very useful and believed that their mentor specifically was very useful in their development as a researcher. One trainee met their mentor daily, two trainees met their mentor weekly, one met their mentor monthly, and one met their mentor daily while in the United States, followed by correspondence by weekly conference calls and emails when they returned to India.

#### Level 2: Learning

The five trainees were required to write a research protocol that modelled a R21 grant application to the NIH. Four of five trainees thought this was very useful, while one thought it was moderately useful. Three trainees thought the mandatory ethics module was very useful, while two thought it was moderately useful. Four trainees thought the courses taken at the University of Pittsburgh were very useful, and one thought it was slightly useful.

#### Level 3: Behaviour

All five of the trainees reported that they use the skills they learned in the programme in their current research. Three trainees stated that they obtained a new research job or acquired a higher position after the programme. Two trainees stated that they obtained a research grant after the programme. Four published at least one manuscript. Four built connections with mentors and one built connections and collaborated with other trainees. Three trainees stated that the programme deepened their interest in psychiatric genetics. Three trainees said their current research is very aligned with the research proposed or performed in the programme, while one said it was moderately aligned. One trainee did not respond to the survey. Additionally, four of the trainees in this cohort became mentors to trainees of other training programmes.

#### Level 4: Results

The trainees in this group (*N* = 5) have published 77 papers for a total of 104 citations, 26 (25%) of which were first author citations by these trainees.

### The TNTPPG

Four trainees completed the TNTPPG. Two trainees were from India and two were from Egypt. All four trainees responded to the Qualtrics survey.

#### Level 1: Reaction

All four trainees thought the training programme was very useful and stated that their mentor had been very useful in their development as a researcher. Three trainees met their mentors daily and one met their mentor weekly.

#### Level 2: Learning

Three out of four trainees thought the PhD protocol they wrote was very useful for their learning, while one thought it was moderately useful. All four trainees stated that the ethics module was very useful. Three trainees stated that the courses they took at the University of Pittsburgh were very useful, while one thought it was only slightly useful.

#### Level 3: Behaviour

Three out of four trainees stated that they use the skills they learned in the programme in their current research and had published at least one publication as a result of being in the programme. Two trainees obtained a new research job or higher position, built connections with mentors and other trainees, and deepened their interest in psychiatric genetics. One trainee stated their that research was very aligned with the research proposed or performed during the programme, while three trainees stated that their research is moderately aligned with their research done in the programme.

#### Level 4: Results

The four trainees in this group have been published in13 manuscripts, 9 (69.2%) of which are first-author citations. These papers include citations for trainees in the TPPG and CFRT Program.

### CFRT for new investigators in Egypt and India

As of 2019, during the preparation of this manuscript, this training programme was still in progress. The first three classes had 38 medium-term trainees enrolled, of which 33 completed the programme. There were 4 long-term trainees enrolled in the programme. Qualtrics surveys were sent to all 38 initially enrolled trainees and all four long-term trainees. Of these, 28 (74%) medium-term trainees responded to the survey and 2 (50%) long-term trainees responded to the survey. Thus, 30 (71%) trainees in total responded to our inquiry. Additionally, 15 of 21 (71%) mentors in this training programme responded to our survey.

#### Level 1: Response – trainees

Overall, 28 (93%) of trainees who responded indicated that the programme was very or moderately useful and 2 (7%) stated that the programme was slightly useful. There was a wide range of responses in the level of communication between mentors and trainees. Eight (27%) of the mentees stated that they only had contact with their mentor every few months, 2 (7%) had contact with their mentor monthly, 5 (17%) had contact weekly, 4 (13%) had contact every few days, and 6 (20%) had contact with their mentor daily. Twelve of the 30 (40%) stated their mentor was very useful in their development as a researcher, 10 (33.3%) stated their mentor was moderately useful, 6 (20%) stated their mentor was slightly useful, and 2 (7%) stated their mentor was not useful in their development as a researcher. In all, 10 (33.3%) trainees stated the most beneficial part of the training programme was the protocol that they had to write.

#### Level 1: Response – mentors

Eleven of the 15 mentors (73%) who completed the surveys thought that the programme was very useful, while 4 (27%) found the programme to be moderately useful. Six (40%) mentors stated that one of the most beneficial aspects of the programme was the exposure to research, 3 mentors (20%) stated that networking with other researchers was most beneficial and 2 (13%) stated that grant writing practice was the most important part of the programme for their mentees. The most frequent improvement mentors requested was to create more structure in the relationship between mentors and trainees in terms of the amount of communication and create definite expectations of the mentors.

#### Level 2: Learning – video lectures

Trainees were asked to watch a series of videotaped lectures that focused on a variety of research skills. Each video was roughly an hour long and there were 13 videos. Twenty-four of the 30 (80%) trainees who responded said the quizzes accurately represented their knowledge of the material, while 5 (16%) said it did not. One respondent did not answer the questions. When questioned how much time trainees spent on videos, the responses had a wide range. Four (13%) spent more than 20 hours watching the videos, 7 (23%) spent between 16 and 20 hours, 9 (30%) spent 11–15 hours, 6 (20%) spent 6–10 hours, 2 (7%) spent 1–5 hours, and 2 (7%) did not watch the videos. Trainees listed which videos were the most beneficial to their learning; 19 (63%) trainees selected the ‘writing grant proposals and manuscripts’ and ‘critical appraisal’ videos, 18 (60%) trainees selected ‘systematic retrieval using PubMed’, 14 (47%) selected ‘protocol development’, 12 (40%) selected ‘epidemiology’, 10 (33%) selected ‘ethical foundation of research’ and ‘biostatistics’, 9 (30%) selected ‘data management’ and ‘writing informed consent forms’, 8 (27%) selected ‘formulation of clinical questions’ and ‘oral presentations’, and 5 (16%) selected ‘challenges of informed consent’ and ‘recruitment and retention of participants’.

In addition, these trainees completed pre- and post-tests to demonstrate changes in knowledge related to the online video lectures. Pre- and post-data were available for 29 of the 30 trainees. There was a 14% overall improvement in knowledge scores overall. A paired-samples *t*-test demonstrated a statistically significant difference in pre- versus post-test scores (*t* (28) = − 8.547, *p* < 0.001).

#### Level 2: Learning – additional learning requirements

Participants were required to write a sample research protocol in NIH format. Seventeen (57%) found this exercise very useful, 7 (23%) found it moderately useful, 5 (17%) found it slightly useful, and 1(3%) did not respond to this question. In regards to the ethics module, 16 (53%) found it very useful, 6 (20%) found it moderately useful, 6 (20%) found it slightly useful, and 1 (3%) did not find it useful.

#### Level 3: Behaviour

Twenty-four (80%) of the trainees reported that they use the skills learned in the programme in their current research; 17 (57%) indicated that they have deepened their interest in interventional schizophrenia treatment as a result of the training programme; 14 (47%) reported that they have built lasting connections with their mentors; 10 (33%) have been published and 8 reported grant funding as a result of their participation in training; 7 (23%) have built connections with other trainees; and 5 (17%) reported a new or higher research position. Out of the medium-term trainees, 8 (29%) stated that their current research was very aligned with their research ideas in the programme, 9 (32%) said it was moderately aligned, 4 (14%) said it is slightly aligned, and 5 (17.9%) said it is not aligned. Two respondents (7%) did not answer the question.

#### Level 4: Results

These training cohorts have published 45 papers and 63 citations, 23 (36.5%) of which as first authors.

##### ICMR workshop

The ‘Grantathon’ with ICMR resulted in five multi-site and seven single-site research proposals with 24 Principal Investigators (three pairs on same projects) in all, which were funded as ICMR Task Force proposals and were currently ongoing at the time of this writing.

##### IAPP workshop

Because this training was provided in response to Indian psychiatrists in private practice and was not part of our overall training approach, participants in the IAPP workshop were only assessed on Level 4 outcomes: Results. A total of five psychiatrists participated in the IAPP workshop. As of the writing of this manuscript, two participants from this group had seven citations, one (14.3%) of which as a first-author. This workshop resulted in two research proposals, one of which was funded by the training programme and the other by the IAPP.

### Overall publications

In total, these 66 trainees have published 130 papers with 187 citations, 59 (31.6%) of which as first-authors. Manuscripts were published in a number of international journals, including *Schizophrenia Research* (Impact Factor 4.748), *American Journal of Medical Genetics* (Impact Factor 9.924) and *Biological Psychiatry* (Impact Factor 11.501). There was some overlap in co-authored manuscripts by trainees across the training programmes. Of the 70 papers authored by TPPG trainees, 5 were co-authored by TNTPPG trainees and 2 by CFRT trainees. In turn, one paper was co-authored by a CFTP and TNTPPG trainees.

**Fig. 1:**
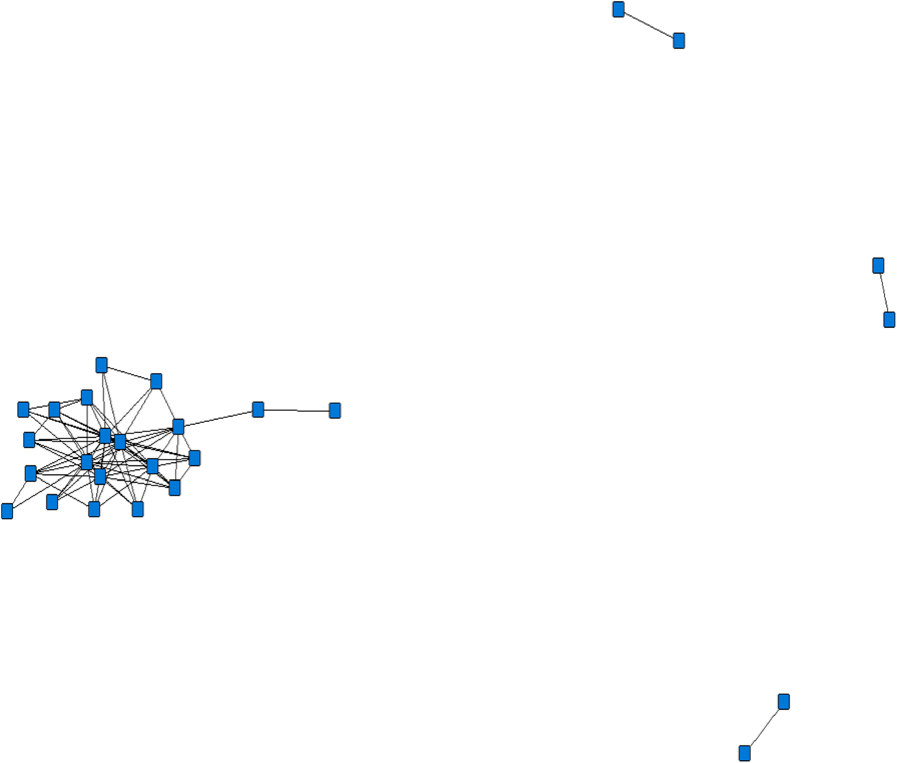
Authorship network analysis

We also conducted a social network analysis using UCINET [21] to explore collaborations among trainees. Figure 1 represents a plot of co-authorship amongst trainees and mentors, with each square or ‘node’ representing an individual author and each line or ‘tie’ representing co-authorship among authors. Only those authors with at least one co-authorship with another trainee or mentor are represented here (*n* = 26). Each line on the graph represents that two individuals co-authored at least one manuscript; therefore, lines indicate only co-authorship presence and not the total number of co-authored papers by authors. The centre of the figure shows denser ties, representing the individuals with the most author collaborations. One trainee had the highest number of collaborations at 14. The three sets of ties on the right side of the figure show three pairs, where each author in each pair co-authored only with one other. In total there are a total of 144 collaborative relationships shown here.

## Discussion

Our findings present opportunities to refine existing training programmes and may benefit other organisations interested in similar approaches. The use of the Kirkpatrick model provides insight as to the trainees’ steps toward independent mental health research. Overall, trainees across all three programmes reported that their training experiences improved their knowledge of research and helped them to publish manuscripts and obtain independent research funding. It is also worth noting that mentors responded positively to the training, with 15 of 21 mentors from the United States, Egypt and India having completed mentor surveys, all of whom indicated that they felt the training was moderately (*n* = 4) or very (*n* = 11) useful. Many mentors are still in touch with trainees, providing ongoing mentorship and guidance.

Given the relatively recent participation of trainees in these research training activities, we may not have observed the full extent and impact of the training. Earlier trainees have obtained more grants and publications to date than the more recent trainees. Qualtrics surveys will be sent out to the final class of medium-term trainees to track the research productivity of the trainees and progress in their careers.

The training programmes also strengthened collaborations with local institutions, specifically ICMR and IAPP. Both organisations requested additional capacity-building trainings, as reported above. The ICMR-sponsored Grantathon workshop resulted in the successful funding of 12 single- or multi-site research projects, which were in their second year of implementation as this manuscript was being prepared. These projects provide mental health interventions addressing a range of concerns, including suicide, mental and physical health co-morbidities, and mental distress in regions under political conflict. Two workshops evaluating the progress of these projects have been further conducted. Representatives of the ICMR, principal investigators and mentors together evaluated the ongoing projects. All PIs found the ongoing oversight very helpful and constructive.

Our findings should be interpreted in view of several limitations. In the CFRT grant, a class of medium-term trainees has been selected every year for the past few years. Thus, trainees in the first two classes have completed the programme years ago. Therefore, a significant amount of time has passed between completion of the programme and completion of the Qualtrics survey, possibly introducing recall bias to results. Trainees who finished the programme earlier had more time to publish their papers or obtain a grant than those that just recently finished. Additionally, five medium-term trainees who had initially enrolled but did not complete the programme were also asked to complete the survey. As the responses to the survey are anonymous, it is uncertain whether they completed the survey, although IP addresses were checked to ensure each trainee only answered once. Long-term trainees had not completed their training programmes at the writing this paper. Lastly, not all trainees answered the surveys.

Still, our findings are useful in several ways. First, feedback from trainees enables opportunities for programme improvement. Specifically, we will update our video lecture library and improve our pre- and post-tests to gain a deeper understanding of how didactic learning opportunities contribute to trainees’ knowledge. In addition, we will implement clearer guidelines and expectations for communication between mentors and mentees. Given the important role of mentors in the development of trainees toward research independence, adding structure to this element of training is likely to create even stronger results.

## Conclusion

The Indo–United States–Egypt tri-national psychiatric research training programmes suggest a successful model for international collaboration. These research training programmes have allowed new researchers to increase their exposure and experience with research in psychiatric disorders, a field with a high need for quality research. While most doctoral programmes expect students to publish manuscripts, there are disparities across these programmes regarding access to publishable data, intensive writing mentorship, and the opportunity to co-author with internationally respected psychiatric researchers. Our training programmes aimed to supplement and fill gaps in these traditional training programmes. Many of the manuscripts published by these researchers included several trainees and mentors as co-authors. The collaborative nature of these publications increases the reach of the trainees and their research findings. In the future, we will continue to expand our research-based training programmes to include topics on cognitive dysfunction in other severe mental illnesses beyond schizophrenia as well as post-stroke cognitive disability. We will also expand our training to other medical specialties, add implementation research training, and broaden our reach beyond individuals to academic institutions and non-governmental organisations through ‘hub building’ work.

Bolstering health research capacity will enable LMICs to pinpoint interventions that are effective in their cultural and geographic settings, to apply and replicate results, and ultimately to strengthen mental health service systems. Unfortunately, in many LMICs, including India, mental health research has lagged behind. Mentoring, training and cultivating the next generation of researchers is a critical step towards the development of effective interventions and increasing expertise in regions with high burdens of mental health need.

## Data Availability

All data generated or analysed during this study are included in this published article.

## Abbreviations

CFRT: Cross-Fertilized Research Training
IAPP: Indian Association of Private Psychiatry
ICMR: Indian Council of Medical Research
LMICs: low- and middle-income countries
NIH: National Institute of Health
TNTPPG: Tri-National Training Program for Psychiatric Genetics
TPPG: Training Program for Psychiatric Genetics
